# Partition Coefficients (*logP*) of Hydrolysable Tannins

**DOI:** 10.3390/molecules25163691

**Published:** 2020-08-13

**Authors:** Valtteri Virtanen, Maarit Karonen

**Affiliations:** Natural Chemistry Research Group, Department of Chemistry, University of Turku, FI-20014 Turku, Finland; maarit.karonen@utu.fi

**Keywords:** ellagitannins, gallic acid derivatives, gallotannins, HPLC, hydrolysable tannins, hydrophobicity, octanol-water partition coefficient, structure-activity, UPLC

## Abstract

The partition coefficients (*logP*) between *n*-octanol and water of 47 purified and characterized hydrolysable tannins were measured with the shake flask method utilizing UPLC and HPLC with UV detection. Results show that galloyl glucoses and gallotannins are clearly more hydrophobic than ellagitannins but the differences in hydrophobicity within ellagitannins are more varied than within galloyl glucoses or gallotannins. Most notable structural features that were found to influence the hydrophobicity of ellagitannins were the number of free galloyl groups, acyclic versus cyclic polyol, substitution of the anomeric position of glucose and ^4^C_1_ versus ^1^C_4_ conformation of the glucopyranose core.

## 1. Introduction

Tannins are specialized plant metabolites, which are typically classified into three main subgroups based on their different structural features i.e., hydrolysable tannins (HTs), proanthocyanidins and phlorotannins. HTs have been shown to possess many nutritionally and pharmacologically beneficial activities such as oxidative activity, protein precipitation capacity and anthelmintic activity [1,2,3,4,5,6]. However, the hydrophobicities of these compounds have not been studied as much as the aforementioned activities even though it is likely one of the main factors when determining the mechanisms of their biological activity, lipid affinity and metabolism in biological systems. Hydrophobicity is usually estimated with partition coefficient measurements utilizing a solvent pair such as *n*-octanol/water [7]. Similar partitioning processes happen all the time in biological systems in vivo for example when compounds are transported into cells [8]. This makes partition coefficient a very suitable predictor when estimating the probability for a compound to reach the desired destination efficiently like for example if it would be absorbed from the gut of an animal. Currently there are only a few studies concerning the hydrophobicity of HTs most probably due to the extensive work of isolating and purifying these compounds and because of how time-consuming accurate partitioning coefficient measurements with the well-established shake-flask method are. Tanaka et al. measured the partition coefficient of 21 HTs which already showed some interesting structure-activity patterns concerning the hydrophobicity of galloyl glucoses and ellagitannins [9]. Additionally, Mueller-Harvey et al. measured the partition coefficient of several tannin rich plant extracts and a few purified tannins [10]. The aim of this study was to expand the knowledge on the hydrophobicity of HTs with a larger set of purified and characterized HTs than before in order to reveal and understand the structural features affecting their hydrophobicity which would help in estimating the biological activity of these compounds better. 

## 2. Results and Discussion

### 2.1. Hydrolysable Tannins Studied

**Figure 1:**
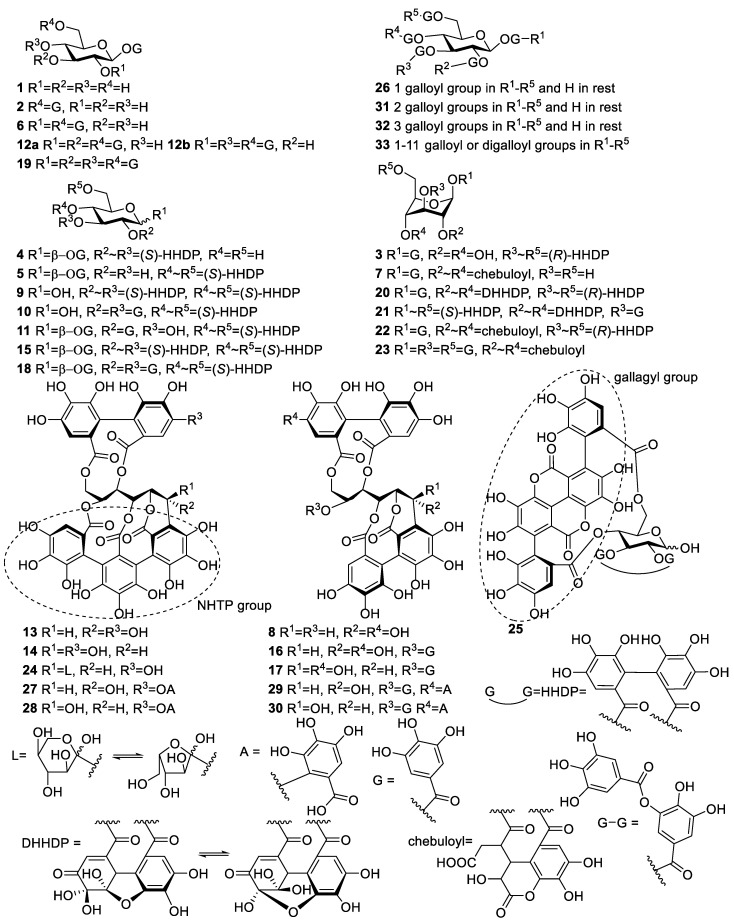
Chemical structures of monomeric hydrolysable tannins used in the study. DHHDP = dehydrohexahydroxydiphenoyl, A = gallic acid, G = galloyl, HHDP = hexahydroxydiphenoyl, L = lyxose, NHTP = nonahydroxytriphenoyl. **1** 1-*O*-galloyl-β-d-glucose, **2** 1,6-di-*O*-galloyl-β-d-glucose, **3** corilagin, **4** isostrictinin, **5** strictinin, **6** 1,2,6-tri-*O*-galloyl-β-d-glucose, **7** chebulanin, **8** casuariin, **9** pedunculagin, **10** tellimagrandin I, **11** 1,2,-di-*O*-galloyl-4,6-HHDP-β-d-glucose, **12a** 1,2,3,6-tetra-*O*-galloyl-β-d-glucose, **12b** 1,2,4,6-tetra-*O*-galloyl-β-d-glucose, **13** castalagin, **14** vescalagin, **15** casuarictin, **16** casuarinin, **17**, stachyurin, **18** tellimagrandin II, **19** 1,2,3,4,6-penta-*O*-galloyl-β-d-glucose, **20** geraniin, **21** carpinusin, **22** chebulagic acid, **23** chebulinic acid, **24** grandinin, **25** punicalagin, **26** hexagalloylglucose, **27** castavaloninic acid, **28** vescavaloninic acid, **29** hippophaenin B, **30** hippophaenin C, **31** heptagalloylglucose, **32** octagalloylglucose, **33** gallotannin mixture. Actual galloylation location of compounds **26**, **31**, **32** and **33** were not confirmed.

**Figure 2:**
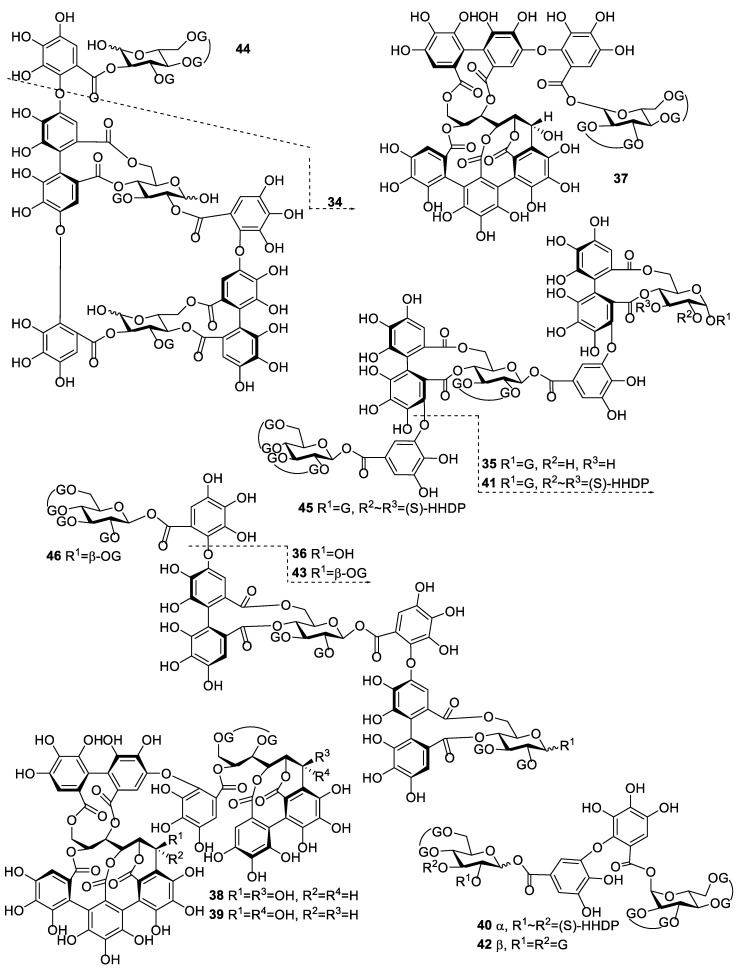
Chemical structures of oligomeric hydrolysable tannins used in the study. **34** oenothein B, **35** rosenin C, **36** rugosin E, **37** cocciferin D-2, **38** salicarinin A, **39** salicarinin B, **40** agrimoniin, **41** sanguiin H-6, **42** gemin A, **43** rugosin D, **44** oenothein A, **45** lambertianin C, **46** rugosin G. See Figure 1 for the details of substitute groups.

We isolated, purified and studied the partition coefficients of 47 individual HTs (Supplementary Material Table S1). The HTs studied in this work were selected to represent the whole HT group as extensively as possible. In general, HTs can be further divided into simple gallic acid derivatives, gallotannins (GT) and ellagitannins (ET) [11]. All simple gallic acid derivatives in this study were glucose based, i.e., they were galloyl glucoses (GGs). From these HT subgroups, ETs are the most varying structurally, which is why they are typically further categorized into simple HHDP-esters, dehydro-ellagitannins, modified dehydro-ellagitannins, C-glycosidic ellagitannins and flavono-ellagitannins. Another level of complexity is added to ET oligomers that differ based on the types of linkages between the monomeric units. Figure 1 shows all the monomeric HTs used in the study and all the possible repeated substitute groups found in their structures. Likewise, oligomeric HTs are presented in Figure 2. Altogether, the studied group of HTs consisted of 6 galloyl glucoses, 4 gallotannins, 9 simple HHDP-esters, 10 C-glycosidic ellagitannins, 2 dehydro-ellagitannins, 3 modified dehydro-ellagitannins and 13 ellagitannin oligomers.

### 2.2. Hydrophobicity of Ellagitannins from Partition Coefficient Measurements

**Figure 3:**
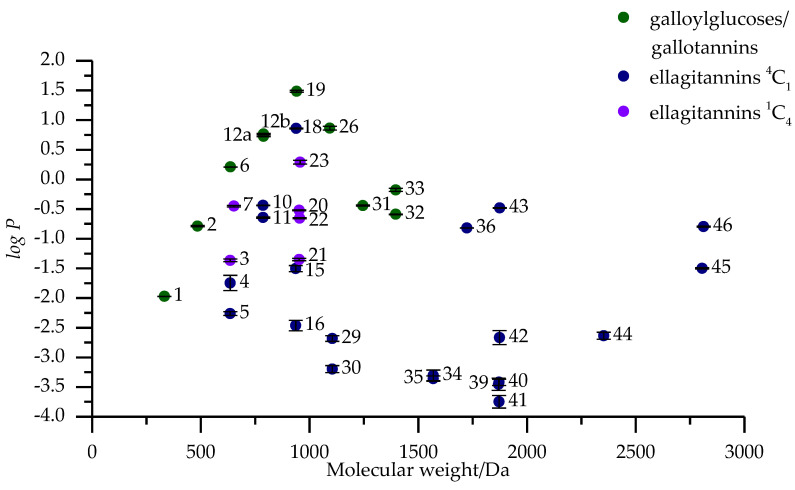
*LogP* values of 47 hydrolysable tannins plotted against their molecular weights measured with UPLC showing galloylglucoses/gallotannins, ^4^C_1_ glucose core ellagitannins and ^1^C_4_ glucose core ellagitannins in different series. The numbers refer to Figure 1 and Figure 2 and Table A1.

Octanol-water partition coefficient (*logP*) is determined as the decadic logarithm of the ratio of the concentration of analyte in the octanol phase to the concentration of analyte in the water phase [7]. The analysis of partition coefficient measurements was done with both HPLC and UPLC with three replicates. Both methods used the same reversed-phase chromatography to make the results comparable and the usage of two analysis methods proved useful when we were able to get a result for some HTs only with UPLC. In some instances, partition coefficient measurements are done so that only a sample from one of the phases and the original sample are analyzed and then the content of the unanalyzed phase is calculated as their difference without regarding the recovery of the analyzed compound due to poor solubility or other issues. Therefore, we analyzed the original sample and samples from both the water and octanol phases. By analyzing all three samples we could be sure what the recovery rate of our HT was after the phase separation and how much the HT had transitioned to either phase accurately. Results from the partition coefficient measurements by UPLC are presented in Figure 3, which will be used/referenced in the following structure-activity pattern evaluations. Table A1 contains the measured *logP* values for both HPLC and UPLC. All the *logP* values used as examples in the forthcoming evaluations are only from the UPLC measurements as the correlation between the *logP* values of HPLC and UPLC analyses was high with an R^2^ value of 0.99.

#### 2.2.1. Galloylglucoses and Gallotannins

**Figure 4:**
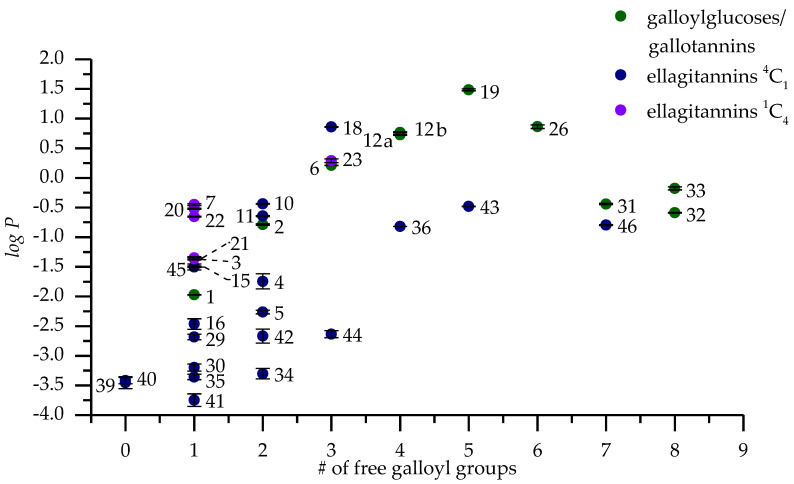
*LogP* values measured with UPLC and plotted against the number of free galloyl groups in the structures of hydrolysable tannins. The numbers refer to Figure 1 and Figure 2 and Table A1.

The *logP* of the measured galloylglucoses (**1**, **2**, **6**, **12a**, **12b**, **19**) increases almost linearly (R^2^ = 0.98) against the degree of galloylation (Figure 4) from 1-*O*-galloyl-β-d-glucose (**1**) all the way until 1,2,3,4,6-penta-*O*-galloyl-β-d-glucose (**19**) with the linearity diminishing when approaching **19**, which clearly follows the same trend also seen in Tanaka et al. [9]. The difference in the location of galloylation in the glucose core of 1,2,3,6-tetra-*O*-galloyl-β-d-glucose (**12a**) and 1,2,4,6-tetra-*O*-galloyl-β-d-glucose (**12b**) did not have a profound effect on their *logP* values (0.77 ± 0.01 vs. 0.73 ± 0.01) which suggests that the substitution of positions 3 and 4 of the glucose core have only a nominal effect in hydrophobicity. On the other hand in Tanaka et al., they had included 2,3,4,6-tetra-*O*-galloyl-β-d-glucose in their study, which had a much smaller *logP* than 1,2,3,6-tetra-*O*-galloyl-β-d-glucose (**12a**) in comparison [9]. This suggests that the substitution of anomeric position 1 of glucose has a much larger impact on the hydrophobicity than other positions. The importance of the anomeric position of glucose has also been witnessed in other HT structure-activity relationship studies before but mainly with the configuration of the anomeric hydroxyl group in C-glycosidic ellagitannins regarding their oxidative activity, protein precipitation capacity and how efficiently they effect the eff hatching and motility of *Haemonchus contortus* larvae [1,2,5].

When 1,2,3,4,6-penta-*O*-galloyl-β-d-glucose (**19**) is galloylated further into hexa- (**26**), hepta- (**31**) and octagalloylglucoses (**32**) forming digalloyl or even trigalloyl groups the hydrophobicity of the compounds decreases almost as drastically as it rises in the GG series. However, the trend of the decline seems to plateau towards octagalloylglucose (**32**). Unfortunately, we did not have the possibility to measure the *logP* of larger purified gallotannins to see how the trend would have continued. Instead we had a gallotannin mixture (**33**) fractionated by Sephadex LH-20 chromatography that consisted of hexa- to hexadecagalloylglucoses with percentage contributions of each gallotannin based on the integrated areas of [M − 2H]^2−^ ions in electrospray ionization mass spectrometry (ESI-MS) being: hexa (16.25%); hepta (19.01%); octa (17.24%); nona (14.72%); deca (12.58%); undeca (9.39%); dodeca (5.94%); trideca (3.05%); tetradeca (1.22%); pentadeca (0.40%); hexadeca (0.12%). These values are more to give an overview to what the GT composition is approximately like than as exact quantitative values due to the possible fragmentation in the ion source and the different efficiencies of ionization of different sized GTs. The GT mixture had a higher *logP* than octagalloylglucose and even higher than heptagalloylglucose but that could be explained by the high amount of hexagalloylglucose in the mixture, which was shown to have a *logP* so high that it alone probably influences the measured *logP* so much that the contribution of the larger GTs remains uncertain.

#### 2.2.2. Ellagitannins with ^4^C_1_ Glucopyranose Cores

**Figure 5:**
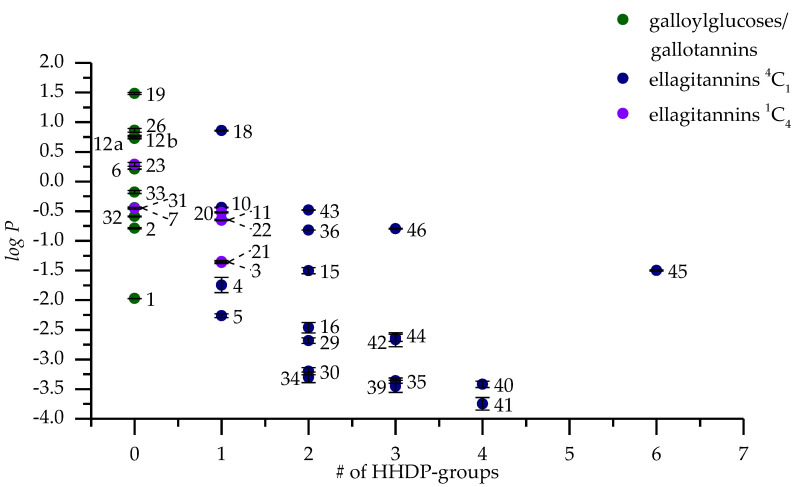
*LogP* values measured with UPLC and plotted against the number of (*S*)- and (*R*)-hexahydroxydiphenoyl (HHDP) groups in the structures of hydrolysable tannins also counting the HHDP groups participating in *m*-GOD (sanguisorboyl)- and *m*-DOG (valoneoyl)-type oligomeric linkages or in valoneoyl units in hippophaenins (**29** and **30**), castavaloninic acid (**27**) and vescavaloninic acid (**28**). Results follow the same trends seen also in Tanaka et al. [9]. The numbers refer to Figure 1 and Figure 2 and Table A1.

ETs having (*S*)-HHDP groups formed from adjacent galloylgroups showed in all cases smaller *logP* values than their GG counterparts. The distinction between S and R configurations of HHDP groups is made here because most of the ETs that are formed first in the biosynthetic pathway of HTs have the glucose polyol in ^4^C_1_ configuration and almost exclusively the HHDP groups in S configuration [12,13]. The reverse is also true in most cases, i.e., ^1^C_4_ ETs have (*R*)-HHDP groups although there are some exceptions like for example carpinusin (**21**) that has an (*S*)-HHDP group even though the glucose is in ^1^C_4_ configuration. The impact of the (*S*)-HHDP group on the hydrophobicity is easiest seen by comparing compounds 1,2,3,4,6-penta-*O*-galloyl-β-d-glucose (**19**), tellimagrandin II (**18**) and casuarictin (**15**). **18** and **15** both have an (*S*)-HHDP group in O4~O6 of glucose while **15** has an additional (*S*)-HHDP group in O2~O3. **19** had the highest *logP* of all the HTs measured in this study (1.49 ± 0.02), while the *logP* of **18** and **15** were smaller (0.86 ± 0.01; −1.50 ± 0.05, respectively). This comparison (Figure 5) shows that while the formation of the first (*S*)-HHDP group had a decreasing effect to the hydrophobicity of the compound, the formation of the second (*S*)-HHDP group had an even greater decreasing effect. This is probably partially explained by the effect of rigidity the second HHDP group has on the chemical structure and the decrease of the number of freely rotating galloylgroups from three in **18** to one in **15**. When the last free galloylgroup in O1 of glucose is absent like in the case of pedunculagin (**9**, Table A1), the compound was so water soluble that it did not transfer to the octanol phase at all during the partition coefficient measurements so a *logP* value could not be determined.

The same trend of (*S*)-HHDP groups decreasing the hydrophobicity of HTs was witnessed with tellimagrandin I (**10**) and 1,2-di-*O*-galloyl-4,6-HHDP-β-d-glucose (**11**) when compared against either of the tetragalloylglucoses (**12a** and **12b**). **10** and **11** are positional isomers where the diverging galloyl group in **10** is in O3 of glucose whereas in **11** it is in O1 of glucose. Unexpectedly, **11** had a lower *logP* value than **10** (−0.64 ± 0.01 vs. −0.44 ± 0.01) even though it had a galloyl group in O1 of glucose, which is perceived to be one of the more important substitution positions regarding the bioactivity of HTs. The definitive reason behind this result could not be determined only based on the compounds used in this study.

The location of the (*S*)-HHDP group has a clear impact on the hydrophobicity of ETs, which can be seen from the *logP* values of isostrictinin (**4**) and strictinin (**5**). **4** and **5** are also positional isomers where the former has an (*S*)-HHDP in O2~O3 and the latter in O4~O6. *LogP* of **4** was measured to be larger than that of **5** (−1.75 ± 0.13; −2.26 ± 0.03, respectively) which indicated that the (*S*)-HHDP in O2~O3 increased the hydrophobicity of the HT compared to when it was in O4~O6. This can be partially explained by the fact that when the HHDP group is in O2~O3, the methylene group in the C6 of glucose is left free which increases hydrophobicity compared to when the HHDP group is in O4~O6 and the C2 and C3 have hydroxyl groups [14].

#### 2.2.3. Ellagitannins with ^1^C_4_ Glucopyranose Cores

As mentioned previously, when ellagitannins are formed from galloylglucoses, the polyol glucose is typically in ^4^C_1_ configuration. If these ETs are biosynthesized further via enzymatic reactions, the HHDP groups can oxidize into dehydrohexahydroxydiphenoyl (DHHDP) groups (Figure 1). One of the most common substitution positions for these DHHDP groups is in the O2~O4 of glucose, which might strain the cyclic glucose and be the reason that these dehydroellagitannins typically have the energetically unfavorable ^1^C_4_ configuration. Corilagin (**3**) is a similar galloyl-HHDP-glucose like isostrictinin (**4**) and strictinin (**5**), however, its glucose is in the ^1^C_4_ configuration. The *logP* value of **3** (−1.36 ± 0.02) was measured to be higher than that of either **4** or **5**. The higher hydrophobicity of **3** cannot be solely attributed to the configuration of the glucose because it isn’t the only structural difference even between ETs **3** and **5** as the HHDP group is in O3~O6 of glucose in the former and in O4~O6 in the latter. In addition, the HHDP group in **3** is in R configuration compared to the S in **4** and **5**.

Geraniin (**20**) and carpinusin (**21**) are both dehydroellagitannins of the same molecular weight where the former has an (*R*)-HHDP group in O3~O6 of glucose and a galloyl group in O1 and the latter has an (*S*)-HHDP group in O1~O6 of glucose and a galloyl group in O3. **20** had a much higher *logP* value than **21** (−0.52 ± 0.01 vs. −1.35 ± 0.02). The free galloyl group in the anomeric position of **20** is probably one of the largest contributors to this increased hydrophobicity but the differences in the substitution and configurations of HHDP group might also have their own effect and cannot be disregarded. Both **20** and **21** have higher hydrophobicity than the similar sized ET with a ^4^C_1_ glucopyranose core, i.e., casuarictin (**15**), which has two HHDP groups instead of the former pairs HHDP and DHHDP groups but still lower than **18**, which has only one HHDP group and three free galloyl groups. This once again demonstrates how much the free galloyl groups can increase the hydrophobicity of HTs compared HHDP and even DHHDP groups.

In addition to the DHHDP group, there is another common oxidized group found in ETs with ^1^C_4_ glucopyranose cores and that is the chebuloyl group (Figure 1) also known as a modified DHHDP group although it is still not clear if this group is directly formed from the DHHDP group [15]. Chebulanin (**7**) is the smallest modified dehydroellagitannin close in molecular size to ETs **3**, **4** and **5** but slightly larger due to the chebuloyl group. The *logP* value of **7** (−0.45 ± 0.01) is the highest of these four ellagitannins but still not as high as that of 1,2,6-tri-*O*-galloyl-β-d-glucose **(6)**. Based on these ETs, the chebuloyl group increases the hydrophobicity more than either the (*S*)- or (*R*)-HHDP groups but still not as much as free galloyl groups. Chebulagic acid (**22**) and chebulinic acid (**23**) are also modified dehydroellagitannins structurally similar to the ETs **20** and **21** (Figure 1). The measured *logP* value of **22** (−0.65 ± 0.01) was lower than that of **20**, which suggests that the DHHDP is a more hydrophobic group than the chebuloyl group due it being the only difference between these compounds. When the HHDP group in **22** was replaced with two galloyls the *logP* of **23** (0.29 ± 0.03) was measured to be much higher than any of the other ETs with a ^1^C_4_ glucopyranose core. Notably, chebulinic acid proved so hydrophobic that its recovery was 41.6% (Table A1) even when the phases were diluted after phase separation as described in Section 3.5.

#### 2.2.4. C-glycosidic Ellagitannins

Most C-glycosidic ETs have an α- and β-anomer, referring to the configuration of anomeric position of their open chain glucose. In a previous study on HT structure-activity relationships, the α-anomer has been shown to be more active than the β-anomer [5]. In addition, the α-anomer elutes always later than the β-anomer in reversed-phase chromatography (Table A1: α vs. β; **13** vs. **14**; **16** vs. **17**; **27** vs. **28**; **29** vs. **30**), which already suggests that the α-anomer is more hydrophobic because non-polar compounds elute later in reversed-phase LC. Out of the ten measured monomeric C-glycosidic ETs, seven (**8**, **13**, **14**, **17**, **24**, **27** and **28**) were so hydrophilic that during the measurements they transferred completely to the water phase and as a result their *logP* values could not be determined (Table A1). The exceptions to this were casuarinin (**16**), hippophaenin B (**29**) and hippophaenin C (**30**), which all had low *logP* values. Structurally all three are similar as they have one free galloyl group attached to O5 of glucose and two (*S*)-HHDP groups in O1~O2 and O3~O6; in addition, **29** and **30** have an additional gallic acid attached to the latter HHDP group. **16** showed the highest *logP* value (−2.46 ± 0.09) over **29** (−2.68 ± 0.05) and **30** (−3.20 ± 0.06). Interestingly, the free acid group seems to have an increasing effect on the hydrophobicity of a β-anomer because **30** has a measurable *logP* when compared to β-anomer of **16** i.e., **17,** which was too hydrophilic even though **16** showed a higher *logP* than **29**. As expected, *logP* of **29** was higher than that of its β-anomer **30** and similarly, **16** showed a measurable *logP* whereas its β-anomer **17** did not. However, it is noteworthy that structural hydrophobicity deductions are challenging based on as small differences in as low *logP* values as witnessed with these three compounds. The lower the *logP* value is the smaller the measured UV area is in the octanol phase which makes even a slight difference seem larger when presented as the logarithm.

Castalagin (**13**) and vescalagin (**14**) have very rigid structures due to the nonahydroxytriphenoyl group at C1~O2~O3~O5 of glucose and the (*S*)-HHDP group at O4~O6 and also highly hydrophilic (Table A1). Castavaloninic acid (**27**) and vescavaloninic acid (**28**) have the same core structure as **13** and **14** but the (*S*)-HHDP group in O4~O6 is substituted with a gallic acid, but the effect of this substitution could not be determined because all four were too hydrophilic for a measurable *logP*. When a lyxose unit is added to the structure of **14** forming grandinin (**24**), the hydrophobicity wasn’t increased measurably, which was expected because, for example, in flavonoid glycosides additional carbohydrate units decrease their hydrophobicity [16]. Casuariin (**8**) has the same structure as **16** except instead of the free galloyl group in O5 of glucose there is a hydroxyl group, which made the ET so hydrophilic that no *logP* could be measured. Another way to evaluate the hydrophilicity of **8** is to compare it against pedunculagin (**9**) which is also a bis-HHDP-glucose but glucopyranose based. In general, most of the glucopyranose based ETs seem to be more hydrophobic than the C-glycosidic ones, which suggests that if **9** was too hydrophilic to transfer to the octanol phase (Table A1) then **8** could not be more hydrophobic.

#### 2.2.5. Oligomeric Ellagitannins

The hydrophobicity of HT monomers differs drastically from those of the oligomers the same monomeric units can form. We measured three different oligomeric HT series containing a monomer, a dimer and a trimer: I) tellimagrandin I (**10**); oenothein B (**34**), oenothein A (**44**); II) casuarictin (**15**), sanguiin H-6 (**41**), lambertianin C (**45**); III) tellimagrandin II (**18**), rugosin D (**43**), rugosin G (**46**). Series I differs from the other two in that the dimer (**34**) is macrocyclic meaning that the monomeric units are attached via two *m*-DOG (valoneoyl)-type linkages, which makes the formed dimers structure more rigid when compared to the other two dimers that only have one bond between the monomers. The trimer (**44**) however is formed by attaching the monomeric unit via only one *m*-DOG-type linkage, which makes the structural progression of series I nonlinear. In the second series oligomers, one of the monomeric units in dimer and trimer is the α-anomer of **15** but otherwise they are formed by adding another monomer via an *m*-GOD (sanguisorboyl)-type linkage. The series III is formed straightforwardly by adding monomeric units via an *m*-DOG-type linkage.

A clear trend could not be detected in the hydrophobicities of the oligomeric series. In all three series the hydrophobicity decreases immensely when moving from the monomer to dimer but comparatively more in series I and II than in III. The hydrophobicity of the trimers in series I and II is higher than their respective dimers but the series III trimer has a lower hydrophobicity than its dimer. This might be due to **43** already having a higher hydrophobicity than even the monomeric structures of series I and I. In series I and II, the dimers on the other hand had so low hydrophobicities that even the increased molecular size of the trimers might increase their hydrophobicity. The highest hydrophobicity out of the dimers of **43** can be partially explained by the five free galloyl groups in its structure in comparison to two free galloyl groups in **34** and one free galloyl group in **41**. Surprisingly, **34** had a higher hydrophobicity than **41** even though it has a rigid macrocyclic structure, however, it seemed that the free galloyl groups it has in both of the monomeric units outweigh this when compared against **41**, which only has one free galloyl group.

Rosenin C (**35**) is similar in size to **34** but very different in structure (Figure 2). **35** does not have a macrocyclic structure, which should make it more flexible when compared to **34** but still both showed relatively similar hydrophobicities. This suggests that the formation of the macrocyclic structure has a similar decreasing effect on the hydrophobicity as the increase of HHDP groups. Last cyclic ET oligomers that produced measurable *logP* values were agrimoniin (**40**) and gemin A (**42**). When compared them against similar sized dimers **41** and **43,** it can be seen again how the number of free galloyl groups affects the hydrophobicity (Figure 4). The difference in the configuration of the anomeric position of some of the monomeric units is not remarked here. The change in *logP* values of these four dimers (**40**–**43**) can be seen in Figure 4 to increase when the number of galloyl groups is increased with the exception of **41** for some reason having an even smaller *logP* than **40,** which had no free galloyl groups. This may be due to the different type of linkage between the monomeric units where **40** has a *m*-GOG (dehydrodigalloyl)-type linkage which should be very flexible compared against the DOG-type linkage in **41**. However, when the number of free galloyl groups is increased beyond the first one into two in **42** and five in **43**, the hydrophobicity clearly increases as seen also with the monomers previously. One of the unusual ET dimers, cocciferin D_2_ (**37**), has one cyclic and one acyclic monomeric unit in its structure and the addition of this acyclic castalagin (**13**) type monomer seems to make it so hydrophilic that the *logP* value could not be measured. However, salicarinin B (**39**) is a completely acyclic dimer with only one free galloyl group that still had a measurable *logP* which was almost as high as that of the dimer with cyclic monomers (**40)**.

## 3. Materials and Methods

### 3.1. Chemicals

Technical grade acetone was purchased from VWR (Haasrode, Belgium). Analytical grade acetone, analytical grade methanol, HPLC gradient grade acetonitrile, HPLC gradient grade methanol, HPLC grade phosphoric acid, HPLC grade formic acid and LC-MS grade formic acid were purchased from VWR International (Fontenay-Sous-Bois, Paris, France). HPLC grade *n*-otanol and LC-MS grade acetonitrile were purchased from Merck (KGaA, Darmstadt, Germany). Aa grade ethanol (≥99.5%) was purchased from Altia OyJ (Helsinki, Finland). Ultra-pure type I water was purified with Merck Millipore Synergy UV system.

### 3.2. Isolation of Hydrolysable Tannins

Original plant material of willow herb flowers (*Epilobium angustifolium*), purple loosestrife leaves (*Lythrum salicaria),* English oak acorns (*Quercus robur*), meadowsweet flowers (*Filipendula ulmaria*), raspberry leaves (*Rubus idaeus*), herb bennet leaves (*Geum urbanum*), silverweed leaves (*Potentilla anserine*) and wood cranesbill leaves (*Geranium sylvaticum*) were collected during years 2011–2018 from the surrounding areas of Turku Finland. Black myrabolan (*Terminalia chebula*) leaf powder was purchased from Banyan Botanicals (Albuquerque, NM). Sea Buckthorn (*Hippophae rhamnoides*) leaf powder was purchased from All-Russian Institute of Medicinal and Aromatic Plants (Moscow, Russia). 1,2,3,4,6-penta-*O*-galloyl-β-d-glucose (**19**) was prepared from tannic acid as described in Salminen and Lempa [17]. The extraction of plant material and the isolation of HTs was performed as previously described [2,3,18,19,20,21,22]. Shortly, plant material was collected in 1 L bottles, which were filled with acetone and placed in 4 °C for maseration. After maseration, the acetone extract was collected, new acetone/water (4:1, *v*/*v*) was added and maseration was continued. This step was repeated multiple times after, which all extracts were combined, concentrated to water phase and lyophilized. Extracts were first crudely fractionated with Sephadex LH-20 gel material in a large beaker using water, methanol/water (1:1, *v*/*v*) and acetone/water (4:1, *v*/*v*) as the elution solvents [21]. Subsequent fractionation was done in a glass column loaded with Sephadex LH-20 gel, which was dissolved and stabilized in water. Typical fractionation protocol was similar to the one described in Salminen and Karonen [20] but for some fractionations, it was modified in order to achieve better separation for compounds that would co-elute with the normal protocol. Final purifications were done with preparative and semipreparative liquid chromatography as described in Karonen et al. [21]. All purification steps and purities of final products were followed with UPLC-DAD-ESI-MS.

### 3.3. Characterization of Isolated Hydrolysable Tannins

Isolated HTs (Supplementary Materials Table S1) were characterized based on their elution order in reverse-phase LC, UV spectra, molecular ions and characteristic fragment ions based on our previous work [1,5,19,23,24,25,26] and references therein. In addition, the NMR spectra of **4**, **6**, **7**, **8**, **12a**, **12b**, **24, 37** and **46** were measured to verify their structure and match the assignations found in literature [27,28,29,30,31,32,33,34]. Measurements were done with either the Bruker Avance-III 500 spectrometer equipped with a Smartprobe (Fällanden, Switzerland), which was operated at 500.08 MHz for ^1^H and 125.76 MHz for ^13^C or the Bruker Avance-III 600 spectrometer equipped with Prodigy TCI (inverted CryoProbe) cooled via liquid nitrogen, which was operated at 600.16 MHz for ^1^H and 150.19 MHz for ^13^C-NMR data and the corresponding ^1^H and ^13^C assignations are presented in Supplementary Material Tables S2–S12 and Figures S1–S9. Typical ^1^H, ^13^C-NMR and several 1D-TOCSY (total correlation spectroscopy) spectra were recorded for all, in addition to multiple 2D spectra including DQF-COSY (double-quantum filtered correlation spectroscopy), ROESY/NOESY (rotating-frame nuclear Overhauser effect spectroscopy/nuclear Overhauser effect spectroscopy), CH_2_-edited HSQC (heteronuclear single quantum coherence) and HMBC (heteronuclear multiple bond correlation). Measurements were done at 25 °C in acetone-*d6*.

### 3.4. HPLC-DAD and UPLC-DAD Analyses

The HPLC-DAD used in all analyzes was a Merck Hitachi (Merck KGaA, Darmstadt; Hitachi Instruments, Inc, San Jose, CA, USA) instrument consisting of a pump, an auto sampler, a column, a DAD detector and an interface module. Column used was Acclaim^®^ Polar Advantage II (C18, 4.6 × 150 mm, 3 μm; Dionex Bonded Silica Products, Sunnyvale, CA, USA). Mobile phase consisted of acetonitrile (A) and 0.05 M aqueous phosphoric acid (B) with a constant flow rate of 1 mL min^−1^ with the following gradient: 0–5 min: 5% A; 5–30 min: 5–37% A (linear gradient); 30–32 min: 37–70% A (linear gradient; 32–40 min: 70% A; 40–43 min: 70–5% A (linear gradient); 43–58 min: 5% A. UV–Vis data were recorded from 0 to 58 min at 195–450 nm wavelength range. The injection volume was 5 µL. The UPLC used in all analyses was an Acquity UPLC (Waters Corp., Milford, MA, USA) instrument consisting of a binary solvent manager, a sample manager, a column and a diode array detector. The column used was an Acquity BEH phenyl column (2.1 × 100 mm, 1.7 µm; Waters Corp., Wexford, Ireland). Mobile phase consisted of acetonitrile (A) and 0.1% aqueous formic acid (B) with a constant flow rate of 0.5 mL min^−1^ with the following gradient: 0–0.5 min: 0.1% A; 0.5–5.0 min: 0.1–30% A (linear gradient); 5.0–6.0 min: 30–35% A (linear gradient); 6.0–6.1 min: 35–90% A (linear gradient): 6.1–8.1 min: 90% A; 8.1–8.2 min: 90–0.1% A (linear gradient); 8.2–9.5 min: 0.1% A. UV–Vis data were recorded from 0 to 7 min at 190–499 nm wavelength range. The injection volume was 5 µL.

UPLC-DAD-ESI-MS analyses for purified HTs were done with two UPLC-MS systems both equipped with the same type of UPLC as explained above. Routine measurements were done with a Xevo TQ triple-quadrupole mass spectrometer (Waters Corp., Milford, MA, USA) coupled to the UPLC via an ESI source. Accurate mass measurements were done with a Q Exactive hybrid quadrupole-Orbitrap mass spectrometer (Thermo Fisher Scientific GmbH, Bremen, Germany) coupled to the UPLC via a heated ESI source.

### 3.5. Partition Coefficient Measurements

Partition coefficient measurements were done with the shake-flask method as follows: Both the *n*-octanol and water phases were saturated with each other in large glass bottle in a planar shaker overnight and the phases were then separated before each day’s measurements. Appropriate amount of the studied HT was weighed in a 2 mL Eppendorf tube for either 1mM or 0.5 mM solutions. This sample was dissolved in 2 mL of *n*-octanol saturated water forming the original sample. Then, 500 µL aliquots were separated into three Eppendorfs, 500 µL of water saturated *n*-octanol was added into each replicate, mixtures were vortexed for 120 min and centrifuged for 10 min with 14,000 rpm. After this, the phases were separated and a sample for LC was taken from the original pure HT, the *n*-octanol phase and the water phase of each replicate. For most HTs, these samples were then analyzed as such with the HPLC and a 10-fold dilution was made, using the same solvents i.e., water saturated *n*-octanol for the octanol phase and vice versa for the water phase, for the UPLC analysis in order to achieve a suitable concentration for the detector. However, for HTs that were already known to be highly hydrophobic and thus hard to keep soluble in water either based on literature or on their relatively late retention time in reversed-phase LC, the dilution for both HPLC (2-fold) and UPLC (10-fold) had to be made using ethanol: *n*-octanol (3:7, *v*/*v*) or ethanol:water (3:7, *v*/*v*) solutions for the appropriate phases and the original sample. This ensured that the HTs that were separated into phases were kept soluble for the whole time it took both instruments to finish the measurements. Samples were filtered using a 0.2 µM PTFE-filter before analysis.

### 3.6. Data Analysis and Software

HPLC measurements were carried out and quantitated using the D-7000 HPLC System Manager software Version 3.1 (Merck, KGaA, Darmstadt, Germany; Hitachi Instruments, Inc., San Jose, CA, USA). UPLC measurements were carried out using MassLynx software version V4.2 SCN982 (Waters Corp., Milford, MA, USA) and quantitations made with subprogram TargetLynx XS application manager. Accurate mass measurements were done with Thermo Xcalibur version 4.1.31.9 (Thermo Fisher Scientific Inc., Waltham, MA, USA). NMR data were measured and analyzed using TopSpin software versions 3.5 pl 7 and 3.5 pl 5 (Bruker, Billerica, MA, USA). Graph visualizations were done with Origin 2016 (64-bit) software version SR2 b9.3.2.303 (OriginLab, Northampton, MA, USA).

## 4. Conclusions

**Figure 6:**
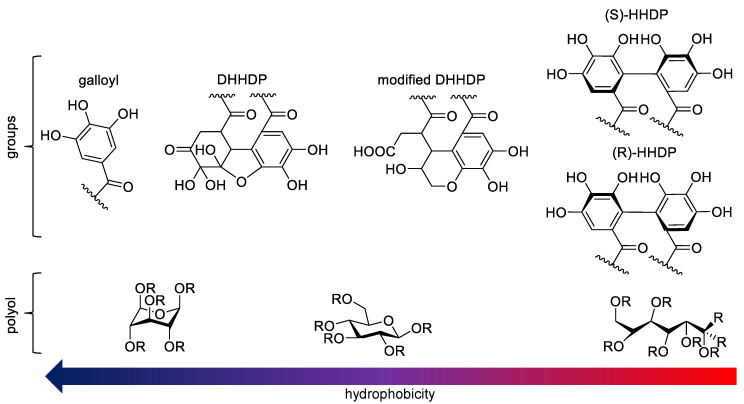
Graphic example of the effects of structural groups and the conformation of the polyol glucose of different hydrolysable tannins on the hydrophobicity. DHHDP = dehydrohexahydroxydiphenoyl, HHDP = hexahydroxydiphenoyl. R can be for instance hydrogen, hydroxyl, galloyl, HHDP or other ET substitute group.

Hydrophobicity is perceived to be one of the essential physicochemical properties that effects how a compound interacts with lipids and permeates cell membranes which is why the structure-activity patterns detected in this study are a solid foundation for more accurate predictions of HT lipid affinities and interactions than were possible with prior knowledge. Based on the different structural comparisons, the following features were determined to be the most dominant factors regarding the hydrophobicity of monomeric HTs (Figure 6). The number of free galloyl groups is the most dominant single factor that increases the hydrophobicity whether the HT is monomeric/oligomeric or cyclic/acyclic. Similarly having a DHHDP group increases the hydrophobicity of a compound more than having a modified DHHDP group and having either one of these increases the hydrophobicity more than having an HHDP group. The difference between the (*S*) and (*R*) configurations of HHDP groups effects on hydrophobicity was not possible to determine with the current structures available. HTs that were glucopyranose based had mainly higher hydrophobicities than C-glycosidic ones possibly due to the very rigid geometries many of these C-glycosidic ETs have compared to the more flexible ones witnessed in glucopyranose ETs. Within the glucopyranose-based ETs, the ones having the energetically unfavorable ^1^C_4_ configuration generally had higher hydrophobicities than the ETs with ^4^C_1_ glucopyranose cores, but this feature was not as significant as the number of free galloyl groups. The substitution of the anomeric position of the polyol glucose also proved to be an important factor in both the glucopyranose-based and C-glycosidic ETs. In glucopyranose-based HTs, simply having a galloyl in the anomeric position rather than in any other position increased the hydrophobicity comparatively more and, in the C-glycosidic ETs, the α-anomer proved to be more hydrophobic than the corresponding β-anomer. The results achieved here provide predictions as to which types of HT structures are more hydrophobic and thus more likely to interact efficiently for example with lipid membranes.

Previously, it has also been hypothesized that the *logP* of a HT could be used as a predictor of their affinity with proteins [10], but this relation might not be this straight forward in all cases. As an example, most of the monomeric ETs that were shown to possess high or even moderate hydrophobicity in this study have also been respectively shown to possess high or moderate affinity to interact or form insoluble complexes with proteins, i.e., the prediction of *logP* works [2,3]. However, almost all oligomeric ETs were shown in this study to have relatively low *logP* values when in Engström et al. many of the same oligomers had much higher capacities to form insoluble complexes with proteins than their monomeric constituents [2]. Nevertheless, ET oligomers that had high *logP* values also had higher capacities to form insoluble complexes with proteins when compared only to other oligomers. This shows that *logP* can be used efficiently as a macromolecule affinity predictor within more homogenic compound groups than the whole hydrolysable tannin group like for example treating HT monomers and oligomers separately.

## Supplementary Materials

The following are available online. Table S1: Purity of hydrolysable tannins studied, English and latin names and plant part used of the original plant material. Figures S1–S9: Chemical structures with numbering and definition of rings of compounds **4**, **6**, **7**, **8**, **12a**, **12b**, **24, 37** and **46**. Tables S2–S12: NMR chemical shifts and coupling constants for compounds **4**, **6**, **7**, **8**, **12a**, **12b**, **24**, **37** and **46**.

## Appendix A

**Table A1:** *LogP* values, standard deviations (SDs) and recovery percentages (rec-%) of 47 hydrolysable tannins (Figure 1 and Figure 2) measured with UPLC and HPLC along with their retention times in UPLC (rt) and molecular weights (MW).

#	rt(UPLC) /min	MW /Da	UPLC			HPLC		
			*logP* ^1^	*logP* SD ^2^	rec-% ^3^	*logP* ^1^	*logP* SD ^2^	rec-% ^3^
1	1.27	332.26	−1.97	0.01	95.0%	−1.92	0.04	100.6%
2	2.80	484.36	−0.79	0.01	90.8%	−0.70	0.01	93.6%
3	3.26	634.45	−1.36	0.02	96.0%	−1.23	0.01	97.6%
4	2.55	634.45	−1.75	0.13	83.4%	−1.62	0.02	99.1%
5	3.11	634.45	−2.26	0.03	98.0%	−2.07	0.08	95.1%
6	3.36	636.47	0.21	0.01	86.8%	0.19	0.01	88.8%
7	3.24	652.47	−0.45	0.01	92.5%	−0.40	0.01	92.0%
8	2.11	784.54	-	-	86.0%	-	-	99.0%
9	2.53/2.85	784.54	-	-	100.5%	-	-	98.4%
10	3.10/3.33	786.55	−0.44	0.01	87.8%	−0.45	0.01	90.8%
11	3.69	786.55	−0.64	0.01	87.6%	−0.61	0.01	92.1%
12a	4.02	788.57	0.77	0.01	79.3%	0.74	0.01	79.4%
12b	4.08	788.57	0.73	0.01	73.6%	0.64	0.01	81.8%
13	2.59	934.63	-	-	100.8%	-	-	100.8%
14	2.23	934.63	-	-	100.3%	-	-	99.0%
15	3.82	936.64	−1.50	0.05	95.4%	−1.22	0.01	97.0%
16	3.20	936.64	−2.46	0.09	97.9%	-	-	97.7%
17	3.07	936.64	-	-	98.7%	-	-	98.0%
18	4.00	938.66	0.86	0.01	92.6%	0.75	0.01	85.9%
19	4.38	940.67	1.49	0.02	77.5%	1.48	0.02	77.6%
20	3.41	952.64	−0.52	0.01	72.8%	−0.39	0.01	82.2%
21	4.01/4.14	952.64	−1.35	0.02	93.8%	−1.20	0.01	92.4%
22	3.87	954.66	−0.65	0.01	92.7%	−0.68	0.01	96.5%
23	4.28	956.67	0.29	0.03	41.6%	0.32	0.05	47.4%
24	2.04	1066.74	-	-	98.0%	-	-	100.3%
25	2.72/3.05	1084.71	-	-	101.3%	-	-	102.2%
26	4.62	1092.78	0.86	0.03	67.7%	0.97	0.03	80.2%
27	2.21	1102.73	-	-	100.5%	-	-	100.7%
28	2.00	1102.73	-	-	100.2%	-	-	98.8%
29	3.18	1104.75	−2.68	0.05	99.6%	−2.17	0.03	95.8%
30	3.03	1104.75	−3.20	0.06	98.2%	-	-	94.7%
31	4.90	1244.88	−0.44	0.01	81.9%	−0.43	0.02	89.8%
32	5.07	1396.99	−0.59	0.01	81.2%	−0.43	0.06	78.8%
33	5.51	1396.99 ^4^	−0.18	0.02	70.7%	−0.01	0.01	88.1%
34	2.95/3.24	1569.08	−3.30	0.09	103.6%	-	-	98.5%
35	3.05	1569.08	−3.36	0.05	98.5%	-	-	95.8%
36	3.86/3.90	1723.20	−0.82	0.00	91.0%	−0.69	0.01	91.9%
37	3.32	1869.25	-	-	95.6%	-	-	92.8%
38	2.28	1869.25	-	-	100.0%	-	-	98.0%
39	2.63	1869.25	−3.45	0.10	98.5%	-	-	102.3%
40	4.16	1871.27	−3.42	0.05	98.5%	-	-	99.4%
41	3.86	1871.27	−3.75	0.11	101.4%	-	-	101.0%
42	4.07	1873.28	−2.67	0.12	103.3%	-	-	98.3%
43	4.22	1875.30	−0.48	0.01	91.3%	−0.49	0.01	91.6%
44	3.28	2353.62	−2.64	0.06	102.7%	-	-	100.3%
45	3.81	2805.90	−1.50	0.01	96.2%	−1.33	0.02	97.3%
46	4.18	2811.94	−0.80	0.01	78.8%	−0.54	0.01	82.7%

^1–3^*LogP* values and their corresponding SDs were calculated as an average of three replicates; ^4^ Molecular weight of the gallotannin mixture presented based on octagalloylglucose.
