# Supplementary material for: Partition Coefficients (logP) of Hydrolysable Tannins
Source: Molecules. 2020 Aug 13;25(16):3691. doi: 10.3390/molecules25163691 (PMC7465006; doi:10.3390/molecules25163691)
Supplement: Supplementary file 1 [file molecules-25-03691-s001.pdf]

SUPPLEMENTARY MATERIALS

# Partition coefficient (*log P*) of hydrolysable tannins

**Valtteri Virtanen [1,*] and Maarit Karonen [1]**

[1] University of Turku, Natural Chemistry Research Group; vtjvir@utu.fi (V.V); maarit.karonen@utu.fi (M.K.)
* Correspondence: vtjvir@utu.fi

# SUPPLEMENTARY MATERIALS CONTENT

**Table S1.** Purity of hydrolysable tannins studied, English and latin names and plant part used of the original plant material.

**Figure S1.** Chemical structure of isostrictinin with numbering and definition of rings.
**Table S2.** NMR chemical shifts and coupling constants for isostrictinin. Measured with Bruker Avance-III 500 spectrometer at 25 °C in acetone-d6.

**Figure S2.** Chemical structure of 1,2,6-tri-*O*-galloyl-β-*D*-glucose with numbering and definition of rings.
**Table S3.** NMR chemical shifts and coupling constants for 1,2,6-tri-*O*-galloyl-β-*D*-glucose. Measured with Bruker Avance-III 500 spectrometer at 25 °C in acetone-d6.

**Figure S3.** Chemical structure of chebulanin with numbering and definition of rings.
**Table S4.** NMR chemical shifts and coupling constants for chebulanin. Measured with Bruker Avance-III 500 spectrometer at 25 °C in acetone-d6.

**Figure S4.** Chemical structure of casuariin with numbering and definition of rings.
**Table S5.** NMR chemical shifts and coupling constants for casuariin. Measured with Bruker Avance-III 500 spectrometer at 25 °C in acetone-d6.

**Figure S5.** Chemical structure of 1,2,3,6-tetra-*O*-galloyl-β-*D*-glucose with numbering and definition of rings.
**Table S6.** NMR chemical shifts and coupling constants for 1,2,3,6-tetra-*O*-galloyl-β-*D*-glucose. Measured with Bruker Avance-III 600 spectrometer at 25 °C in acetone-d6.

**Figure S6.** Chemical structure of 1,2,4,6-tetra-*O*-galloyl-β-*D*-glucose with numbering and definition of rings.
**Table S7.** NMR chemical shifts and coupling constants for 1,2,4,6-tetra-*O*-galloyl-β-*D*-glucose. Measured with Bruker Avance-III 600 spectrometer at 25 °C in acetone-d6.

**Figure S7.** Chemical structure of grandinin with numbering and definition of rings.
**Table S8.** NMR chemical shifts and coupling constants for grandinins main isomer (40.7 %). Measured with Bruker Avance-III 500 spectrometer at 25 °C in acetone-*d6*.
**Table S9.** NMR chemical shifts and coupling constants for grandinins second isomer (34.1 %). Measured with Bruker Avance-III 500 spectrometer at 25 °C in acetone-*d6*.
**Table S10.** NMR chemical shifts and coupling constants for grandinins third isomer (25.2 %). Measured with Bruker Avance-III 500 spectrometer at 25 °C in acetone-*d6*.

**Figure S8.** Chemical structure of cocciferin $D_2$ with with numbering and definition of rings.
**Table S11.** NMR chemical shifts and coupling constants for cocciferin $D_2$. Measured with Bruker Avance-III 600 spectrometer at 25 °C in acetone-d6.

**Figure S9.** Chemical structure of rugosin G with numbering and definition of rings.
**Table S12.** NMR chemical shifts and coupling constants for rugosin G. Measured with Bruker Avance-III 600 spectrometer at 25 °C in acetone-d6.

**Table S1.** Purity of hydrolysable tannins studied, English and latin names and plant part used of the original plant material.

| # | Hydrolysable Tannin | Purity-%[a] | Plant Origin | | |
|---|---|---|---|---|---|
| | | | English Name | Latin Name | Plant Part |
| 1 | 1-*O*-galloyl-β-*D*-glucose | 87.9 % | - | - | - |
| 2 | 1,6-di-*O*-galloyl-β-*D*-glucose | 99.9 % | Meadowsweet | *Filipendula ulmaria* | flower |
| 3 | corilagin | 98.3 % | Black myrabolan | *Terminalia chebula* | leaf |
| 4 | isostrictinin | 93.4 % | Sea buckthorn | *Hippophae rhamnoides* | leaf |
| 5 | strictinin | 98.6 % | Sea buckthorn | *Hippophae rhamnoides* | leaf |
| 6 | 1,2,6-tri-*O*-galloyl-β-*D*-glucose | 93.1 % | Sea buckthorn | *Hippophae rhamnoides* | leaf |
| 7 | chebulanin | 97.0 % | Black myrabolan | *Terminalia chebula* | leaf |
| 8 | casuariin | 92.0 % | Sea buckthorn | *Hippophae rhamnoides* | leaf |
| 9 | pedunculagin | 98.8 % | Silverweed | *Argentina anserina* | leaf |
| 10 | tellimagrandin I | 94.8 % | Meadowsweet | *Filipendula ulmaria* | flower |
| 11 | 1,2-di-*O*-galloyl-4,6-HHDP-β-*D*-glucose | 95.0 % | Meadowsweet | *Filipendula ulmaria* | flower |
| 12a | 1,2,3,6-tetra-*O*-galloyl-β-*D*-glucose[b] | 71.4 % | Norway maple | *Acer platanoides* | leaf |
| 12b | 1,2,4,6-tetra-*O*-galloyl-β-*D*-glucose[b] | 74.7 % | Norway maple | *Acer platanoides* | leaf |
| 13 | castalagin | 99.6 % | English oak | *Quercus robur* | acorn |
| 14 | vescalagin | 97.0 % | English oak | *Quercus robur* | acorn |
| 15 | casuarictin | 97.5 % | Meadowsweet | *Filipendula ulmaria* | flower |
| 16 | casuarinin | 97.5 % | Sea buckthorn | *Hippophae rhamnoides* | leaf |
| 17 | stachyurin | 96.8 % | Sea buckthorn | *Hippophae rhamnoides* | leaf |
| 18 | tellimagrandin II | 90.2 % | Meadowsweet | *Filipendula ulmaria* | flower |
| 19 | 1,2,3,4,6-penta-*O*-galloyl-β-*D*-glucose[b] | 98.6 % | - | - | - |
| 20 | geraniin | 84.8 % | Wood cranesbill | *Geranium sylvaticum* | leaf |
| 21 | carpinusin | 98.3 % | Wood cranesbill | *Geranium sylvaticum* | leaf |
| 22 | chebulagic acid | 96.1 % | Black myrabolan | *Terminalia chebula* | leaf |
| 23 | chebulinic acid | 88.3 % | Black myrabolan | *Terminalia chebula* | leaf |
| 24 | grandinin | 98.3 % | - | - | - |
| 25 | punicalagin | 98.4 % | Black myrabolan | *Terminalia chebula* | leaf |
| 26 | hexagalloylglucose | 94.1 % | Norway maple | *Acer platanoides* | leaf |
| 27 | castavaloninic acid | 91.7 % | English oak | *Quercus robur* | acorn |

| # | Hydrolysable Tannin | Purity-%[a] | Plant Origin | | |
|---|---|---|---|---|---|
| | | | English Name | | |
| 28 | vescavaloninic acid | 97.6 % | English oak | *Quercus robur* | acorn |
| 29 | hippophaenin B | 99.9 % | Sea buckthorn | *Hippophae rhamnoides* | leaf |
| 30 | hippophaenin C | 96.2 % | Sea buckthorn | *Hippophae rhamnoides* | leaf |
| 31 | heptagalloylglucose | 97.2 % | Norway maple | *Acer platanoides* | leaf |
| 32 | octagalloylglucose | 96.8 % | Norway maple | *Acer platanoides* | leaf |
| 33 | gallotannin mixture | 97.8 % | Norway maple | *Acer platanoides* | leaf |
| 34 | oenothein B | 93.1 % | Willowherb | *Chamaenerion angustifolium* | leaf |
| 35 | rosenin C | 94.2 % | Raspberry | *Rubus idaeus* | leaf |
| 36 | rugosin E | 91.2 % | Meadowsweet | *Filipendula ulmaria* | flower |
| 37 | cocciferin $D_2$ | 93.0 % | English oak | *Quercus robur* | acorn |
| 38 | salicarinin A | 96.0 % | Purple loosestrife | *Lythrum salicaria* | leaf |
| 39 | salicarinin B | 75.2 % | Purple loosestrife | *Lythrum salicaria* | leaf |
| 40 | agrimoniin | 97.6 % | Silverweed | *Argentina anserina* | leaf |
| 41 | sanguiin H-6 | 97.1 % | Raspberry | *Rubus idaeus* | leaf |
| 42 | gemin A | 96.9 % | herb Bennet | *Geum urbanum* | leaf |
| 43 | rugosin D | 90.7 % | Meadowsweet | *Filipendula ulmaria* | flower |
| 44 | oenothein A | 84.5 % | Willowherb | *Chamaenerion angustifolium* | leaf |
| 45 | lambertianin C | 98.2 % | Raspberry | *Rubus idaeus* | leaf |
| 46 | rugosin G | 90.3 % | Meadowsweet | *Filipendula ulmaria* | flower- |

[a] Purity measured with the UPLC described in Materials and methods and determined at UV 280±1 nm.

[b] Largest impurity in 1,2,3,6-tetra-*O*-galloyl-β-D-glucose was 1,2,4,6-tetra-*O*-galloyl-β-D-glucose and vice versa.

[c] 1,2,3,4,6-penta-*O*-galloyl-β-D-glucose prepared via methanolysis of tannic acid.

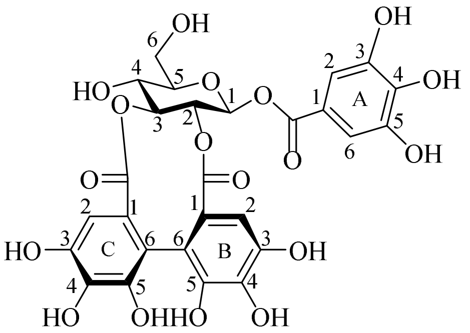


**Figure S1.** Chemical structure of isostrictinin with numbering and definition of rings.

**Table S2.** NMR chemical shifts and coupling constants for isostrictinin. Measured with Bruker Avance-III 500 spectrometer at 25 °C in acetone-*d6*. Assignations match those found in Okuda et al. [1].

| Position | δ $^{13}$C/ppm | δ $^{1}$H/ppm | multiplicity | $J_{H,H}$/Hz |
|---|---|---|---|---|
| 1 | 92.24 | 6.12 | d | 8.50 |
| 2 | 75.51 | 5.00 | t | 9.05 |
| 3 | 80.46 | 5.20 | t | 9.53 |
| 4 | 67.96 | 3.96 | t | 9.55 |
| 5 | 79.05 | 3.73 | ddd | 1.87; 4.37; 9.40 |
| 6 | 61.86 | 3.81 | dd | 1.78; 12.18 |
| | | 3.91 | dd | 4.63; 11.98 |
| C=O (A) | 169.55 | - | - | - |
| A-1 | 120.22 | - | - | - |
| A-2 | 110.26 | 7.16 | s | - |
| A-3 | 146.30 | - | - | - |
| A-4 | 139.75 | - | - | - |
| A-5 | 146.30 | - | - | - |
| A-6 | 110.26 | 7.16 | s | - |
| C=O (B) | 168.85 | - | - | - |
| B-1 | 127.22 | - | - | - |
| B-2 | 114.53 | 6.43 | s | - |
| B-3 | 145.14/145.33[a] | - | - | - |
| B-4 | 136.25 | - | - | - |
| B-5 | 144.42/144.52[b] | - | - | - |
| B-6 | 107.80 | 6.43 | s | - |
| C=O (C) | 165.08 | - | - | - |
| C-1 | 126.69 | - | - | - |
| C-2 | 114.53 | 6.71 | s | - |
| C-3 | 145.14/145.33[a] | - | - | - |
| C-4 | 136.40 | - | - | - |
| C-5 | 144.42/144.52[b] | - | - | - |
| C-6 | 107.24 | 6.71 | s | - |

[a & b] Assignations may be interchangeable and cannot be verified without further measurements.

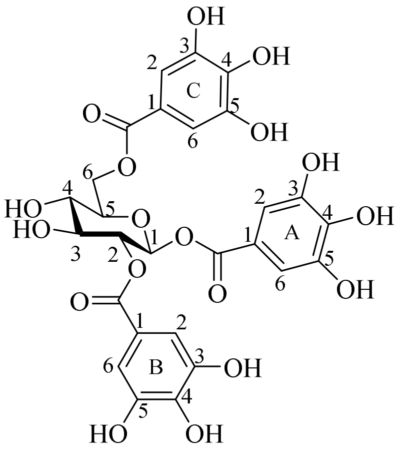


**Figure S2.** Chemical structure of 1,2,6-tri-*O*-galloyl-β-D-glucose with numbering and definition of rings.

**Table S3.** NMR chemical shifts and coupling constants for 1,2,6-tri-*O*-galloyl-β-D-glucose. Measured with Bruker Avance-III 500 spectrometer at 25 °C in acetone-*d6*. Assignations match those found in Nonaka et al. [2].

| Position | δ $^{13}$C/ppm | δ $^{1}$H/ppm | multiplicity | $J_{H,H}$/Hz |
|---|---|---|---|---|
| 1 | 94.08 | 5.99 | d | 8.40 |
| 2 | 74.37 | 5.25 | dd | 8.58; 9.72 |
| 3 | 76.06 | 3.98 | t | 9.20 |
| 4 | 71.63 | 3.76 | t | 9.33 |
| 5 | 76.56 | 3.92 | ddd | 1.88; 4.55; 9.78 |
| 6 | 64.38 | 4.58 | dd | 1.83; 12.13 |
| | | 4.48 | dd | 4.62; 12.13 |
| C=O (A) | 165.61 | - | - | - |
| A-1 | 120.81 | - | - | - |
| A-2 | 110.75 | 7.07 | s | - |
| A-3 | 146.53 | - | - | - |
| A-4 | 140.02 | - | - | - |
| A-5 | 146.53 | - | - | - |
| A-6 | 110.75 | 7.07 | s | - |
| C=O (B) | 166.43 | - | - | - |
| B-1 | 121.98 | - | - | - |
| B-2 | 110.62 | 7.10 | s | - |
| B-3 | 146.41 | - | - | - |
| B-4 | 139.41 | - | - | - |
| B-5 | 146.41 | - | - | - |
| B-6 | 110.62 | 7.10 | s | - |
| C=O (C) | 167.11 | - | - | - |
| C-1 | 122.15 | - | - | - |
| C-2 | 110.45 | 7.15 | s | - |
| C-3 | 146.54 | - | - | - |
| C-4 | 139.37 | - | - | - |
| C-5 | 146.54 | - | - | - |
| C-6 | 110.45 | 7.15 | s | - |

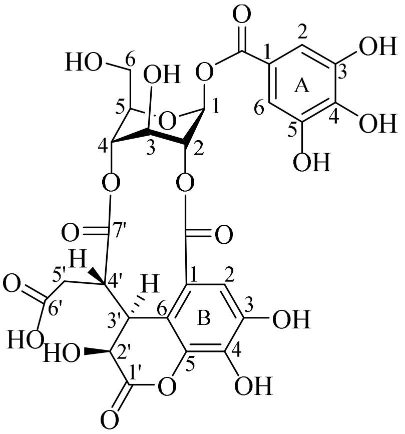


**Figure S3.** Chemical structure of chebulanin with numbering and definition of rings.

**Table S4.** NMR chemical shifts and coupling constants for chebulanin. Measured with Bruker Avance-III 500 spectrometer at 25 °C in acetone-*d6*. Assignations match those found in Luo et al. [3].

| Position | δ $^{13}$C/ppm | δ $^{1}$H/ppm | multiplicity | $J_{H,H}$/Hz |
|---|---|---|---|---|
| 1 | 92.61 | 6.36 | d | 2.85 |
| 2 | 74.07 | 5.24 | brs | - |
| 3 | 61.82 | 4.83 | brs | - |
| 4 | 71.85 | 4.89 | brd | 3.35 |
| 5 | 79.26 | 4.31 | t | 6.00 |
| 6 | 63.26 | 4.14 | brt | 8.20 |
| | | 4.01 | dd | 5.43; 11.08 |
| 1′ | 169.70 | - | - | - |
| 2′ | 66.79 | 4.94 | dd | 3.85; 7.20 |
| 3′ | 41.09 | 5.19 | dd | 1.40; 7.20 |
| 4′ | 39.58 | 3.90 | ddd | 1.34; 4.89; 10.21 |
| 5′ | 30.32 | 2.20 | d | 1.55 |
| | | 2.18 | d | 7.05 |
| 6′ | 172.60[a] | - | - | - |
| 7′ | 174.03[a] | - | - | - |
| C=O (A) | 165.11 | - | - | - |
| A-1 | 120.91 | - | - | - |
| A-2 | 110.40 | 7.20 | s | - |
| A-3 | 146.27[b] | - | - | - |
| A-4 | 139.60[b] | - | - | - |
| A-5 | 146.27 | - | - | - |
| A-6 | 110.40 | 7.20 | s | - |
| C=O (B) | 165.95 | - | - | - |
| B-1 | 119.81 | - | - | - |
| B-2 | 117.17 | 7.50 | s | - |
| B-3 | 146.58 | - | - | - |
| B-4 | 139.30 | - | - | - |
| B-5 | 141.16 | - | - | - |
| B-6 | 116.15 | - | - | - |

[a & b] Assignations may be interchangeable and cannot be verified without further measurements.

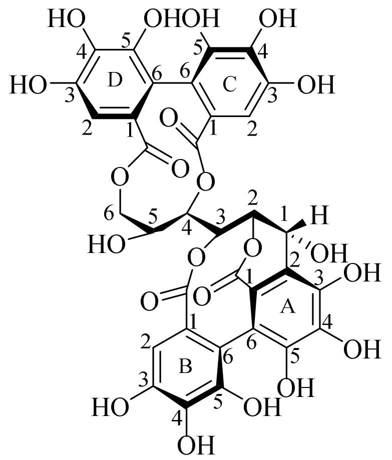


**Figure S4.** Chemical structure of casuariin with numbering and definition of rings.

**Table S5.** NMR chemical shifts and coupling constants for casuariin. Measured with Bruker Avance-III 500 spectrometer at 25 °C in acetone-*d6*. Assignations match those found in Okuda et al. [4].

| Position | δ $^{13}$C/ppm | δ $^{1}$H/ppm | multiplicity | $J_{H,H}$/Hz |
|---|---|---|---|---|
| 1 | 68.67 | 5.65 | d | 4.75 |
| 2 | 77.18 | 4.73 | dd | 2.25; 4.70 |
| 3 | 71.35 | 5.44 | t | 2.45 |
| 4 | 77.87 | 5.00 | dd | 2.78; 8.67 |
| 5 | 68.93 | 4.13 | dd | 2.70; 8.70 |
| 6 | 68.71 | 3.84 | d | 12.20 |
| | | 4.63 | dd | 3.18; 12.33 |
| C=O (A) | 164.40 | - | - | - |
| A-1 | 116.90 | - | - | - |
| A-2 | 116.08 | - | - | - |
| A-3 | 146.89 | - | - | - |
| A-4 | 138.57 | - | - | - |
| A-5 | 144.20 | - | - | - |
| A-6 | 121.61 | - | - | - |
| C=O (B) | 170.83 | 6.43 | s | - |
| B-1 | 116.78 | - | - | - |
| B-2 | 107.36 | - | - | - |
| B-3 | 146.22 | - | - | - |
| B-4 | 135.24 | - | - | - |
| B-5 | 144.54[a] | - | - | - |
| B-6 | 126.17 | - | - | - |
| C=O (C) | 169.10 | 6.66 | s | - |
| C-1 | 116.37 | - | - | - |
| C-2 | 108.59 | - | - | - |
| C-3 | 145.70 | - | - | - |
| C-4 | 137.00 | - | - | - |
| C-5 | 144.95[a] | - | - | - |
| C-6 | 128.19[b] | - | - | - |
| C=O (D) | 169.70 | 6.52 | s | - |
| D-1 | 115.67 | - | - | - |
| D-2 | 105.60 | - | - | - |
| D-3 | 145.55 | - | - | - |
| D-4 | 136.11 | - | - | - |
| D-5 | 145.18[a] | - | - | - |

| | | | | |
|---|---|---|---|---|
| D-6 | 128.37[b] | - | - | - |

[a & b] Assignations may be interchangeable and cannot be verified without further measurements.

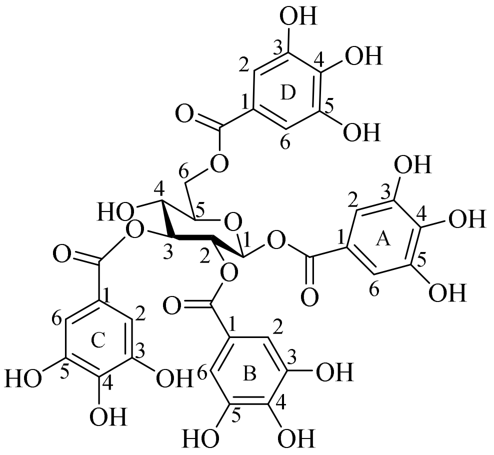


**Figure S5.** Chemical structure of 1,2,3,6-tetra-*O*-galloyl-β-D-glucose with numbering and definition of rings.

**Table S6.** NMR chemical shifts and coupling constants for 1,2,3,6-tetra-*O*-galloyl-β-D-glucose. Measured with Bruker Avance-III 600 spectrometer at 25 °C in acetone-*d6*. Assignations match those found in Haddock et al. [5].

| Position | δ $^{13}$C/ppm | δ $^{1}$H/ppm | multiplicity | $J_{H,H}$/Hz |
|---|---|---|---|---|
| 1 | 93.57 | 6.18 | d | 8.35 |
| 2 | 71.87 | 5.47 | dd | 8.35; 9.90 |
| 3 | 75.98 | 5.67 | dd | 8.90; 9.80 |
| 4 | 69.42 | 4.11 | m | - |
| 5 | 76.14 | 4.13 | m | - |
| 6 | 63.73 | 4.59 | m | - |
| | | 4.59 | m | - |
| C=O (A) | 165.09 | - | - | - |
| A-2 | 110.09/110/166/110.26/110/39[a] | 7.09 | s | - |
| A-6 | 110.09/110/166/110.26/110/39[a] | 7.09 | s | - |
| C=O (B) | 165.84 | - | - | - |
| B-2 | 110.09/110/166/110.26/110/39[a] | 7.00 | s | - |
| B-6 | 110.09/110/166/110.26/110/39[a] | 7.00 | s | - |
| C=O (C) | 166.25 | - | s- | - |
| C-2 | 110.09/110/166/110.26/110/39[a] | 7.07 | s | - |
| C-6 | 110.09/110/166/110.26/110/39[a] | 7.07 | s | - |
| C=O (D) | 166.68 | - | - | - |
| D-2 | 110.09/110/166/110.26/110/39[a] | 7.18 | s | - |
| D-6 | 110.09/110/166/110.26/110/39[a] | 7.18 | s | - |

*Complete $^{13}$C assignation could not be made due to low amount of analyte available.

[a] Assignations may be interchangeable and cannot be verified without further measurements.

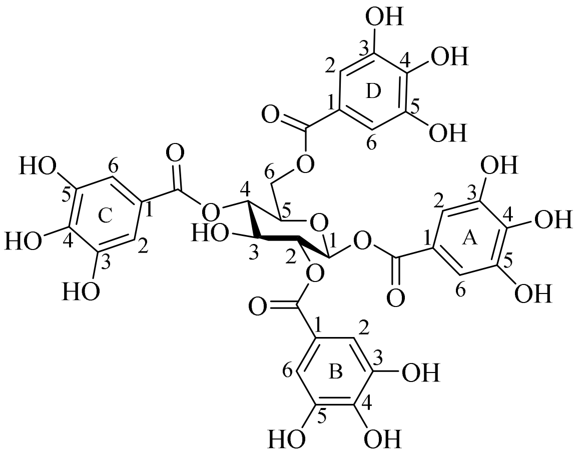

**Figure S6.** Chemical structure of 1,2,4,6-tetra-*O*-galloyl-β-D-glucose with numbering and definition of rings.

**Table S7.** NMR chemical shifts and coupling constants for 1,2,4,6-tetra-*O*-galloyl-β-D-glucose. Measured with Bruker Avance-III 600 spectrometer at 25 °C in acetone-*d6*. Assignations match those found in Haddock et al. [5].

| Position | δ $^{13}C$/ppm | δ $^{1}H$/ppm | multiplicity | $J_{H,H}$/Hz |
|---|---|---|---|---|
| 1 | 93.51 | 6.11 | d | 8.45 |
| 2 | 71.75 | 5.41 | t | 9.50 |
| 3 | 74.14 | 4.31 | m | - |
| 4 | 73.94 | 5.40 | dd | 8.50; 9.50 |
| 5 | 73.45 | 4.39 | td | 5.54; 14.06 |
| 6 | 63.25 | 4.27 | dd | 4.83; 12.03 |
| | | 4.50 | dd | 1.44; 11.94 |
| C=O (A) | 165.17 | - | - | - |
| A-2 | 110.09/110/166/110.26/110/39[a] | 7.09 | s | - |
| A-6 | 110.09/110/166/110.26/110/39[a] | 7.09 | s | - |
| C=O (B) | 165.94 | - | - | - |
| B-2 | 110.09/110/166/110.26/110/39[a] | 7.11 | s | - |
| B-6 | 110.09/110/166/110.26/110/39[a] | 7.11 | s | - |
| C=O (C) | 165.94 | - | - | - |
| C-2 | 110.09/110/166/110.26/110/39[a] | 7.17 | s | - |
| C-6 | 110.09/110/166/110.26/110/39[a] | 7.17 | s | - |
| C=O (D) | 166.53 | - | - | - |
| D-2 | 110.09/110/166/110.26/110/39[a] | 7.16 | s | - |
| D-6 | 110.09/110/166/110.26/110/39[a] | 7.16 | s | - |

*Complete $^{13}C$ assignation could not be made due to low amount of analyte available.

[a] Assignations may be interchangeable and cannot be verified without further measurements.

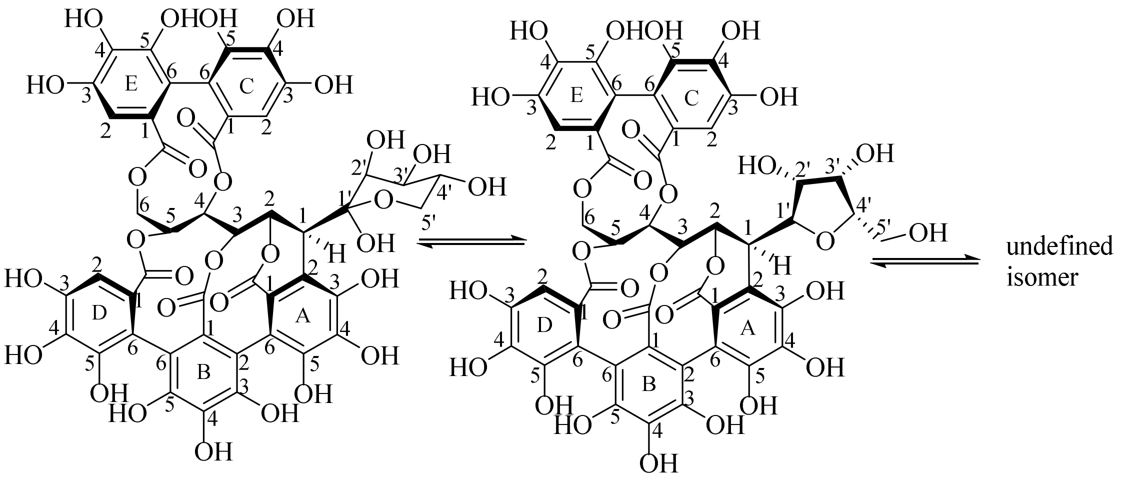


**Figure S7.** Chemical structure of grandinin with numbering and definition of rings.

**Table S8.** NMR chemical shifts and coupling constants for the main isomer of grandinin (40.7 %). Measured with Bruker Avance-III 500 spectrometer at 25 °C in acetone-*d6*. Assignations match those found in Hervé du Penhoat et al. [6].

| Position | δ $^{13}$C/ppm | δ $^{1}$H/ppm | multiplicity | $J_{H,H}$/Hz |
|---|---|---|---|---|
| 1 | 46.81 | 3.55 | brs | - |
| 2 | 72.29 | 5.52 | brs | - |
| 3 | 71.84 | 4.55 | brd | 6.80 |
| 4 | 70.10 | 5.26 | t | 6.98 |
| 5 | 71.38 | 5.65 | brd | 7.05 |
| 6 | 65.67 | 3.99 | d | 12.21 |
| | | 5.02 | dd | 2.30; 12.90 |
| 2′ | - | 4.42 | d | 3.00 |
| 3′ | - | 4.12 | td | 3.37; 9.30 |
| 4′ | - | 4.08 | m | - |
| 5′ | - | 3.81 | d | 10.65 |
| | | 3.86 | dd | 5.60; 10.85 |
| C=O (A) | 164.16 | - | - | - |
| C=O (B) | 165.39/165.49[a] | - | - | - |
| C=O (C) | 166.64/166.62/166.64[b] | - | - | - |
| C-2 | 110.62 | 7.47 | s | - |
| C=O (D) | 166.47/166.53/166.55[c] | - | - | - |
| D-2 | 109.14/109.18/109.23[d] | 6.75 | s | - |
| C=O (E) | 169.07 | - | - | - |
| E-2 | 107.58 | 6.58 | s | - |

* Grandinin occurs as three isomers in solution with the percentage ratios between the isomers being (40.7 %; 34.1 %; 25.2 %). Most probable explanations for two of the isomers are from the pyranose and furanose forms of lyxose but configuration of the last isomer could not be determined. Distinction between which assignated isomer is which could not be made. Complete $^{13}$C assignation could not be made.
[a-d] Assignations could not be separated between the isomers.

**Table S9.** NMR chemical shifts and coupling constants for the second isomer of grandinins (34.1 %). Measured with Bruker Avance-III 500 spectrometer at 25 °C in acetone-*d6*. Assignations match those found in Hervé du Penhoat et al. [6].

| Position | δ $^{13}$C/ppm | δ $^{1}$H/ppm | multiplicity | $J_{H,H}$/Hz |
|---|---|---|---|---|
| 1 | 48.81 | 3.33 | brs | - |
| 2 | 72.48 | 5.68 | brs | - |

| Position | δ ¹³C/ppm | δ ¹H/ppm | multiplicity | $J_{H,H}$/Hz |
|---|---|---|---|---|
| 3 | 71.31 | 4.64 | brd | 7.10 |
| 4 | 69.81 | 5.17 | t | 7.48 |
| 5 | 71.01 | 5.61 | brd | 7.75 |
| 6 | 65.67 | 3.98 | d | 12.51 |
| | | 5.08 | dd | 2.55; 12.95 |
| 2′ | - | 4.24 | d | 4.65 |
| 3′ | - | 4.40 | t | 4.13 |
| 4′ | - | 4.06 | d | 4.50 |
| 5′ | - | 3.91 | d | 5.31 |
| | | 3.91 | d | 6.22 |
| C=O (A) | 164.17 | - | - | - |
| C=O (B) | 165.49 | - | - | - |
| C=O (C) | 166.64/166.62/166.64[a] | - | - | |
| C-2 | 109.14/109.18/109.23[b] | 7.00 | - | - |
| C=O (D) | 166.47/166.53/166.55[c] | - | - | - |
| D-2 | 109.14/109.18/109.23[d] | 6.76 | - | - |
| C=O (E) | 168.91/168.92[e] | - | - | - |
| E-2 | 107.87 | 6.62 | - | - |

* Complete ¹³C assignation could not be made.

a-e Assignations could not be separated between the isomers.

**Table S10.** NMR chemical shifts and coupling constants for the third isomer of grandinin (25.2 %). Measured with Bruker Avance-III 500 spectrometer at 25 °C in acetone-*d6*. Assignations match those found in Hervé du Penhoat et al. [6].

| Position | δ ¹³C/ppm | δ ¹H/ppm | multiplicity | $J_{H,H}$/Hz |
|---|---|---|---|---|
| 1 | 48.68 | 3.37 | brs | - |
| 2 | 71.66 | 5.70 | brs | - |
| 3 | 71.18 | 4.51 | brd | 6.80 |
| 4 | 69.86 | 5.19 | t | 7.38 |
| 5 | 71.01 | 5.61 | brd | 7.85 |
| 6 | 65.67 | 3.98 | d | 12.51 |
| | | 5.12 | dd | 2.70; 13.05 |
| 2′ | - | 4.33 | d | 2.90 |
| 3′ | - | 4.29 | t | 3.03 |
| 4′ | - | 4.01 | d | 3.59 |
| 5′ | - | 3.74 | d | 12.90 |
| | | 4.25 | d | 12.66 |
| C=O (A) | 164.41 | - | - | - |
| C=O (B) | 165.39/165.49[a] | - | - | - |
| C=O (C) | 166.64/166.62/166.64[b] | - | - | - |
| C-2 | 109.2280 | 7.14 | s | - |
| C=O (D) | 166.47/166.53/166.55[c] | - | - | - |
| D-2 | 109.14/109.18/109.23[d] | 6.76 | s | - |
| C=O (E) | 168.91/168.92[e] | - | - | - |
| E-2 | 107.92 | 6.63 | s | - |

* Complete ¹³C assignation could not be made.

a-e Assignations could not be separated between the isomers.

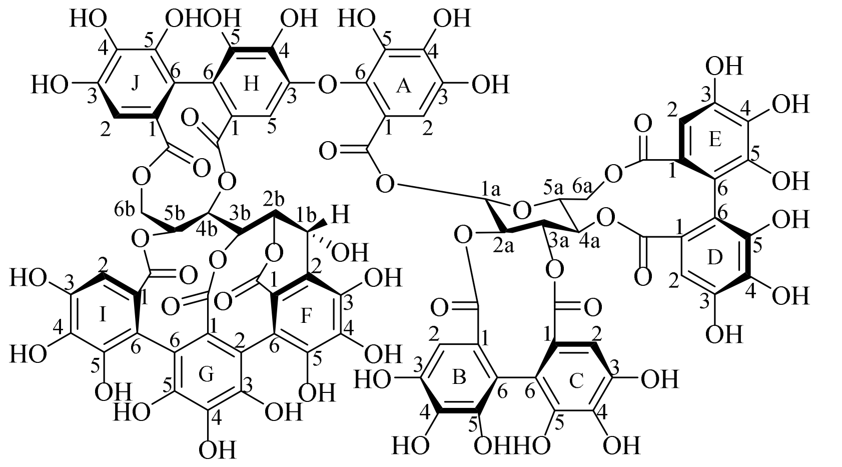

**Figure S8.** Chemical structure of cocciferin $D_2$ with with numbering and definition of rings.

**Table S11.** NMR chemical shifts and coupling constants for cocciferin $D_2$. Measured with Bruker Avance-III 600 spectrometer at 25 °C in acetone-*d6*. Assignations match those found in Ito et al. [7].

| Position | δ $^{13}$C/ppm | δ $^{1}$H/ppm | multiplicity | $J_{H,H}$/Hz |
|---|---|---|---|---|
| 1a | 92.72 | 6.15 | d | 8.55 |
| 2a | 76.47 | 5.14 | t | 10.07 |
| 3a | 77.78 | 5.38 | dd | 9.40; 10.20 |
| 4a | 69.46[a] | 5.13 | t | 8.85 |
| 5a | 73.96 | 4.43 | ddd | 0.89; 6.94; 9.79 |
| 6a | 63.52 | 3.86 | brd | 12.75 |
| | | 5.31 | dd | 6.80; 13.30 |
| C=O (A) | 163.90 | - | -- | |
| A-2 | 110.46 | 7.27 | s | - |
| C=O (B) | 166.39 | - | - | - |
| B-2 | 106.40 | 6.49 | s | - |
| C=O (C) | 169.68 | - | - | - |
| C-2 | 107.85 | 6.35 | s | - |
| C=O (D) | 168.35 | - | - | - |
| D-2 | 109.02 | 6.68 | s | - |
| C=O (E) | 168.29 | - | - | - |
| E-2 | 108.10 | 6.51 | s | - |
| 1b | 67.98 | 5.41 | d | 4.90 |
| 2b | 74.11 | 4.77 | dd | 1.18; 4.88 |
| 3b | 66.79 | 4.91 | dd | 1.05; 6.85 |
| 4b | 69.53[a] | 5.12 | t | 7.32 |
| 5b | 71.85 | 5.55 | dd | 2.18; 7.38 |
| 6b | 65.78 | 3.98 | d | 12.85 |
| | | 5.14 | brd | - |
| C=O (F) | 163.95 | - | - | - |
| C=O (G) | 165.95 | - | - | - |
| C=O (H) | 169.57 | - | - | - |
| H-2 | 108.18 | 6.41 | s | - |
| C=O (I) | 167.00 | - | - | - |
| I-2 | 109.59 | 6.78 | s | - |
| C=O (J) | 169.31 | - | - | - |
| J-2 | 108.31 | 6.59 | s | - |

*Complete $^{13}$C assignation could not be made.

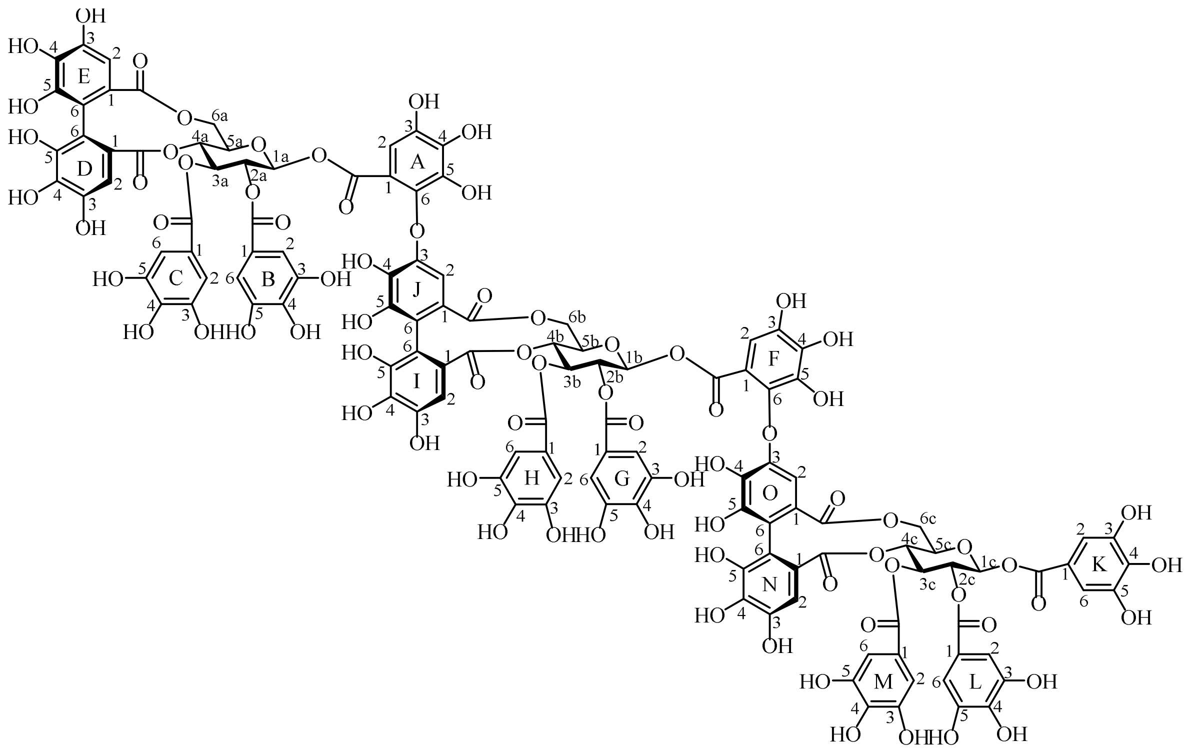

**Figure S9.** Chemical structure of rugosin G with numbering and definition of rings.

**Table S12.** NMR chemical shifts and coupling constants for rugosin G. Measured with Bruker Avance-III 600 spectrometer at 25 °C in acetone-*d6*. Assignations match those found in Ito et al. [8].

| Position | δ $^{13}$C/ppm | δ $^{1}$H/ppm | multiplicity | $J_{H,H}$/Hz |
|---|---|---|---|---|
| 1a | 93.500/93.715/94.140[a] | 6.09 | d | 8.34 |
| 2a | 72.081/72.132[b] | 5.53 | dd | 8.49; 9.45 |
| 3a | 73.642/73.699[c] | 5.78 | t | 9.75 |
| 4a | 71.105/71.140/71.166[d] | 5.09 | t | 9.96 |
| 5a | 73.321/73.414/73.473[e] | 4.43 | dd | 6.78; 10.02 |
| 6a | 63.435[f] | 3.71 | d | 13.32 |
| | | 5.23 | dd | 6.60; 13.50 |
| C=O (A) | 162.618 | - | - | - |
| A-2 | 110.508-110.905[g] | 7.153 | s | - |
| C=O (B) | 166.027/166.040/166.070[h] | - | - | - |
| B-2 | 110.508-110.905[g] | 7.002/7.018/7.026[n] | s | - |
| B-6 | 110.508-110.905[g] | 7.002/7.018/7.026[n] | s | - |
| C=O (C) | 166.651/166.677[i] | - | - | - |
| C-2 | 110.508-110.905[g] | 6.974/6.994/7.006[o] | s | - |
| C-6 | 110.508-110.905[g] | 6.974/6.994/7.006[o] | s | - |
| C=O (D) | 168.112[j] | - | - | - |
| D-2 | 108.126/108.240[k] | 6.462 | s | - |
| C=O (E) | 168.2242[l] | - | - | - |
| E-2 | 105.623[m] | 6.220[c] | s | - |
| 1b | 93.500/93.715/94.140[a] | 6.12 | d | 8.28 |
| 2b | 72.081/72.132[b] | 5.53 | dd | 8.49; 9.45 |
| 3b | 73.642/73.699[c] | 5.78 | t | 9.75 |
| 4b | 71.105/71.140/71.166[d] | 5.16 | t | 10.01 |
| 5b | 73.321/73.414/73.473[e] | 4.47 | dd | 6.99; 9.69 |
| 6b | 63.435[f] | 3.81 | d | 13.32 |

| | | | | |
|---|---|---|---|---|
| | | 5.31 | dd | 6.48; 13.44 |
| C=O (F) | 162.920 | - | - | - |
| A-2 | 110.508-110.905[g] | 7.128 | s | - |
| C=O (G) | 166.027/166.040/166.070[h] | - | - | - |
| B-2 | 110.508-110.905[g] | 7.002/7.018/7.026[n] | s | - |
| B-6 | 110.508-110.905[g] | 7.002/7.018/7.026[n] | s | - |
| C=O (H) | 166.651/166.677[i] | - | - | - |
| C-2 | 110.508-110.905[g] | 6.974/6.994/7.006[o] | s | - |
| C-6 | 110.508-110.905[g] | 6.974/6.994/7.006[o] | s | - |
| C=O (I) | 168.018 | - | - | - |
| C-2 | 108.126/108.240[k] | 6.458 | s | - |
| C=O (J) | 168.396 | - | - | - |
| C-2 | 108.708 | 6.674 | s | - |
| 1c | 93.500/93.715/94.140[a] | 6.19 | d | 8.34 |
| 2c | 72.081/72.132[b] | 5.61 | dd | 8.43; 8.51 |
| 3c | 73.642/73.699[c] | 5.84 | t | 9.75 |
| 4c | 71.105/71.140/71.166[d] | 5.15 | t | 9.93 |
| 5c | 73.321/73.414/73.473[e] | 4.53 | dd | 6.57; 10.05 |
| 6c | 63.435[f] | 3.78 | d | 13.32 |
| | | 5.28 | dd | 6.60; 13.68 |
| C=O (K) | 165.519 | - | - | - |
| A-2 | 110.508-110.905[g] | 7.150 | s | - |
| A-6 | 110.508-110.905[g] | 7.150 | s | - |
| C=O (L) | 166.027/166.040/166.070[h] | - | - | - |
| B-2 | 110.508-110.905[g] | 7.002/7.018/7.026[n] | s | - |
| B-6 | 110.508-110.905[g] | 7.002/7.018/7.026[n] | s | - |
| C=O (M) | 166.651/166.677[i] | - | - | - |
| C-2 | 110.508-110.905[g] | 6.974/6.994/7.006[o] | s | - |
| C-6 | 110.508-110.905[g] | 6.974/6.994/7.006[o] | s | - |
| C=O (N) | 168.112[j] | - | - | - |
| C-2 | 108.126/108.240[k] | 6.474 | s | - |
| C=O (O) | 168.2242[l] | - | - | - |
| C-2 | 105.623[m] | 6.232[c] | s | - |

*Complete $^{13}$C assignation could not be made due to the complexity of the structure.

[a-o] Assignations may be interchangeable and cannot be verified without further measurements.

## References

1. Okuda, T.; Yoshida, T.; Hatano, T.; Yazaki, K.; Ashida, M. Ellagitannins of the casuarinaceae, stachyuraceae and myrtaceae. *Phytochemistry* **1980**, *21*, 2871–2874, doi:10.1016/0031-9422(80)85058-8.
2. Nonaka, G. ichiro; Nishioka, I.; Nagasawa, T.; Oura, H. Tannins and Related Compounds. I.1) Rhubarb (1). *Chem. Pharm. Bull.* **1981**, *29*, 2862–2870, doi:10.1248/cpb.29.2862.
3. Luo, W.; Wen, L.; Zhao, M.; Yang, B.; Ren, J.; Shen, G.; Rao, G. Structural identification of isomallotusinin and other phenolics in Phyllanthus emblica L. fruit hull. *Food Chem.* **2012**, *132*, 1527–1533, doi:10.1016/j.foodchem.2011.11.146.
4. Okuda, T.; Yoshida, T.; Ashida, M.; Yazaki, K. Casuariin, Stachyurin and Strictinin, New Ellagitannins from Casuarina Stricta and Stachyurus Praecox. *Chem. Pharm. Bull.* **1982**, *30*, 766–769.
5. Haddock, E.A.; Gupta, R.K.; Al-Shafi, S.M.K.; Haslam, E.; Magnolato, D. The metabolism of gallic acid and hexahydroxydiphenic acid in plants. Part 1. Introduction. Naturally occurring galloyl esters. *J. Chem. Soc. Perkin Trans. 1* **1982**, 2515–2524, doi:10.1039/P19820002515.
6. Hervé Du Penhoat, C. L. M. Michon, V.M.F.; Peng, S.; Viriot, C.; Scalbert, A.; Gage, D. Structural Elucidation of New Dimeric Ellagitannins from Quercus robur L. Roburins A-E. *J. Chem. Soc. Perkin Trans. 1* **1991**, *53*, 1689–1699, doi:10.1039/P19910001653.
7. Ito, H.; Yamaguchi, K.; Kim, T.H.; Khennouf, S.; Gharzouli, K.; Yoshida, T. Dimeric and trimeric hydrolyzable tannins from Quercus coccifera and Quercus suber. *J. Nat. Prod.* **2002**, *65*, 339–345, doi:10.1021/np010465i.
8. Okuda, T.; Hatano, T.; Ogawa, N. Rugosin D, E, F and G, dimeric and trimeric hydrolyzable tannins. *Chem. Pharm. Bull.* **1982**, *30*, 4234–4237.
